# Hypothyroidism in β-Thalassemia Intermedia Patients with and without Hydroxyurea

**Published:** 2014-01

**Authors:** Omid Reza Zekavat, Ali Reza Makarem, Sezaneh Haghpanah, Zohreh Karamizadeh, Parvin Javad, Mehran Karimi

**Affiliations:** 1 Hematology Research Center, Nemazee Hospital, Shiraz University of Medical Sciences, Shiraz, Iran;; 2 Student Research Committee, Jahrom University of Medical Sciences, Jahrom, Iran;; 3 Department of Pediatric Endocrinology, Nemazee Hospital, Shiraz University of Medical Sciences, Shiraz, Iran

**Keywords:** Thalassemia intermedia, Hydroxyurea, Thyroid function

## Abstract

Hydroxyurea (HU) has been successfully used in patients with β-thalassemia intermedia (β-TI). We aimed to evaluate the effect of the long-term use of HU on thyroid function in patients with β-TI. Seventy-five patients with β-TI aged≥11 years and taking HU were randomly selected during 2010 in southern Iran. Thirty-one patients with β-TI without HU were considered as a control group. Serum levels of thyroid stimulating hormone (TSH) and T4 were measured. The mean age of the participants was 22.7±5.1 years (age range=12-41 years). Serum ferritin level had no significant correlation with HU consumption (P>0.05). Overall, we detected 10 (9.4%) patients with hypothyroidism. We found that the use of HU at a dose of 8-15 mg/kg/day has no significant association with thyroid function in β-TI patients. However, due to the small sample size in our study, documentation of this finding needs further studies with higher numbers of patients.

## Introduction

β-thalassemia intermedia (β-TI) is a term that describes patients with milder anemia than patients with β-thalassemia major.^1^ Ineffective erythropoiesis, chronic hemolytic anemia, and iron overload are the main factors responsible for the disease process in β-TI.^2^Chronic anemia may have such adverse effects as increase in gastrointestinal iron absorption and iron overload, which can cause endocrine abnormalities, diabetes mellitus, osteoporosis, hypothyroidism, and hypogonadism.^3^ Iron chelation therapy, splenectomy, transfusion therapy, and modulation of fetal hemoglobin (HbF) production are several available options for managing patients with β-TI.^2^^,^^3^Pharmacological agents that increase γ-globin production like Hydroxyurea (HU), as evidenced by an increase in HbF, have been considered as therapeutic agents for patients with β-thalassemia.^4^ Increasing the synthesis of fetal hemoglobin can help reduce anemia and, thereby, improve the clinical condition of patients with β-TI.^5^

In several patients with β-TI and in patients with sickle-cell disease, a rise in total HbF level has been repeatedly reported during HU treatment. HU treatment can reduce blood transfusion dependency and even make some patients transfusion free, increasing their energy state and decreasing splenomegaly.^6^HU treatment is protective for hypothyroidism, pulmonary hypertension, extramedullary hematopoiesis, leg ulcers, and osteoporosis.^7^ The commonest side effects of HU therapy include neutropenia and thrombocytopenia, both of which are predictable and easily manageable.^8^

In the few studies conducted on the side effects of HU in β-TI patients, dermatological, neurological, and gastrointestinal adverse effects were seen without any reports of endocrine abnormality, bone marrow suppression, or hematological toxicity.^9^In the present study, medium to long-term follow-up of chronic low-dose HU was inspected to analyze the effect of HU treatment on the thyroid function of patients with β-TI.

## Patients and Methods

This cross-sectional study was done during 2010 in southern Iran. Considering α=0.05, power=70%, and estimated 10% difference of ratio between the two groups, the sample size was calculated as 88 patients by Power SSC software. However, due to financial constraints, we enrolled only 75 patients with β-TI as our case group to be treated with HU. These patients were selected via a simple random sampling method. Diagnosis of β-TI was based on hemoglobin electrophoresis and complete blood count. All the patients were under routine follow-up by an expert hematologist and were blood transfusion independent. Patients with mean serum ferritin level<1000 ng/dl in the recent 5 years, age≥11 years, and HU consumption with a dose of 8-15 mg/kg/day for at least 5 years were included in this study. The control group consisted of 31 patients with β-TI without using HU, ferritin level of <1000 ng/ml (in order to exclude iron overload as a confounding factor) in the recent 5 years, and age≥11 years. The two groups were matched for age and sex. Patients with no desire to participate in the project, ferritin level of >1000 ng/dL in the recent 5 years, or age<11 years were excluded from the study. 

All the patients were referred for paraclinical evaluation, including the serum levels of ferritin, T4, and thyroid stimulating hormone (TSH). Finally, a proficient pediatric endocrinologist reviewed the hormonal profile of the patients to find patients affected by hypothyroidism. The diagnosis of hypothyroidism was based on T4<40 nmol/L and TSH>3.5 µIU/ml. All the laboratory tests were done with the VIDAS PC system and Bio-Merieux kit (Marcy-l Etoile, France). The research protocol was approved by the Deputy Directorship of Research of Shiraz University of Medical Sciences. Informed written consent was obtained from all the adult participants, and for the children, from their parents. 

The data were analyzed by SPSS software (version 17) using the Student *t *test, χ2 test, Fisher exact test, and Pearson correlation test C. A P value<0.05 was considered statistically significant.

## Result

A total of 106 patients with β-TI, comprised of 56 female and 50 male patients with a mean age of 22.7±5.1 years (age range=12–41 years), were included in this study. The range of HU consumption in the patients was between 5 and 13 years. The mean serum ferritin level was 514.5±324.1. Serum ferritin level had no significant correlation with T4 (*r*=-0.185, P=0.059) or TSH (*r*=0.048, P=0.629). There were no statistically significant differences between the case and control groups regarding sex, age, or serum ferritin level (P>0.05). Comparison of T4 and TSH levels between the case and control groups showed no statistically significant differences (P>0.05, table 1). Finally, the hormonal results were classified into two diagnostic categories: euthyroid and hypothyroid groups (table 2). Overall, we detected 10 (9.4%) patients with hypothyroidism. Although the case group had a higher number of patients with hypothyroidism (12% vs. 3%), there was no significant relationship between the use of HU and hypothyroidism (P=0.148). The power of this study was 43%.

**Table 1:** Comparison of serum T4 level and TSH levels as well as serum ferritin level between the patients with β-thalassemia intermedia with and without taking Hydroxyurea (case and control groups)

**HU consumption**	**Age (year)** **mean±SD**	**Ferritin ng/ml** **mean±SD**	**T4 ** **µg/dL ** **mean±SD**	**TSH ** **µIU/ml** **mean±SD**
Without HU N=31	21±3.8	455±296	83±11	2.7±1.7
With HU N=75	23±5.2	538±333	79±13	3.4±2.3

All P values were not significant (>0.05). SD: Standard deviation; HU: Hydroxyurea; TSH: Thyroid stimulating hormone

**Table 2:** Comparison of the frequency of hypothyroidism in the patients with β-thalassemia intermedia with regard to Hydroxyurea consumption

**Patients**	**Euthyroid N (%)**	**Hypothyroidism N (%)**	**Total**
Patients without HU	30 (96.8)	1 (3.2)	31 (100)
Patients with HU	66 (88)	9 (12)	75 (100)

P value=0.148

## Discussion

According to our study, the association between HU consumption and hypothyroidism was not statistically significant (P>0.05).

In patients with β-TM, several endocrine glands may be affected in childhood, adolescence, and adulthood due to iron overload. The Grundy RG et al.^10^ study showed that a tight control of ferritin levels through appropriate chelation does not completely prevent the endocrine (including thyroid) complications**.**^10^ De Sanctis et al.^11^ reported a high prevalence of primary hypothyroidism in β-thalassemia patients with the predominance of its mildest form. A relatively large cohort study by Zarvas A et al.^12^ on 200 thalassemia patients found no significant relationship between ferritin levels and thyroid functional status. In another study by Mariotti et al.^13^ 28.7% of the β-thalassemia patients had primary hypothyroidism (38/132); this was associated with hypoechoic and smaller glands. Taher et al.^14^ study demonstrated that patients with β-TI experience many complications that are thought to be more frequent in β-TI compared with β-TM: thrombosis, extramedullary hematopoiesis, pulmonary hypertension, leg ulcers, and cholelithiasis.

HU is used in β-TI patients in order to decrease the need for transfusion and augment hematological response. Bradai et al.^6^ reported clinical and hematologic improvement with HU and regression of extramedullary hematopoietic masses in patients with β-TI. The literature contains only a few studies on the side effects of HU in β-TI. Ali T. Taher^7^ showed that HU treatment is protective for extramedullary hematopoiesis, pulmonary hypertension, leg ulcers, and hypothyroidism. In our previous study on low-dose HU (mean 10.74 mg/kg/day), adverse effects were recorded in 44 (30.7%) patients. Dermatological side effects, followed by neurological and gastrointestinal adverse effects, were commonly seen without any reports of endocrine abnormality.^9^

We detected a frequency of 10 (9.4%) for hypothyroidism in all our studied β-TI patients. There was no correlation between HU consumption and hormonal disturbance in our patients. To the best of our knowledge, there is no report on the effect of HU on thyroid status in β-TI patients to compare with our result. Patients with β-TI should be meticulously followed up for the early detection and management of newly developed complications.

## Conclusion

In our study, we found that HU at a dose of 8–15 mg/kg/day has no significant association with thyroid function in β-TI patients and it could be used as a safe treatment in these patients. Given the rise in mean HbF levels following HU therapy and decrease in transfusion requirement and iron overload complications like thyroid dysfunction in thalassemia patients, HU therapy may be protective for hypothyroidism. It should be mentioned that our study was limited due to the small number of patients in each group, which shows the need for conducting further studies with higher numbers of patients to find more accurate statistical relationships.
